# Perceived cancer-related stigma among Arab patients: A cross-sectional study

**DOI:** 10.1371/journal.pone.0333185

**Published:** 2025-09-26

**Authors:** Fawwaz Alaloul, Mohammad Al Qadire, Omar Al Omari, Sulaiman Al Sabei, Mudhar Al Adawi, Zamzam Al-Habsi

**Affiliations:** 1 College of Nursing, Sultan Qaboos University, Muscat, Sultanate of Oman; 2 School of Nursing, Al Al-Bayt University, Mafraq, Jordan; 3 Department of Nursing, Royal Hospital, Ministry of Health (MOH), Muscat, Oman; 4 Sultan Qaboos University Hospital, Al Seeb, Oman; Qatar University College of Nursing, QATAR

## Abstract

**Purpose:**

The primary objective of this study was to examine the perceived stigma experienced by patients with cancer within the context of Arabic and Islamic culture.

**Methods:**

A cross-sectional study was conducted with 190 Arab Muslim patients with cancer from two government-designated hospitals. Participants completed the Social Impact Scale, and descriptive statistics were calculated for the four subdomains associated with perceived stigma.

**Results:**

The average stigma score was 82.36, indicating a high level of perceived stigma. The dimensions of stigma, ranked in descending order, were as follows: social rejection (M = 3.69), financial security (M = 3.35), isolation (M = 3.34), and internalized shame (M = 3.16).

**Conclusion:**

The study findings indicate that Muslim Arab patients with cancer experience significant stigmatization. Social rejection was the most significant dimension of stigma, followed by concerns regarding financial security, feelings of isolation, and internalized shame. Culture and religion can play a vital role in influencing perceptions and experiences of cancer, leading to varying levels of stigma associated with the disease across diverse cultural backgrounds.

## Introduction

Stigma is a social phenomenon in which an individual’s condition renders them unaccepted or rejected by the community [1]. Weiss and Ramakrishna [2] defined stigma as a social process or personal experience involving exclusion, rejection, blame, or devaluation. It arises from the actual experience or the rational anticipation of negative social judgment directed at an individual or a collective entity.

Health-related stigmatization is the negative evaluation of a person due to a disease or condition [3]. Individuals facing stigma may encounter different types, including social and self-stigma [4]. Social stigma occurs when community members discredit individuals with specific attributes [4,5]. Self-stigma involves an internalized sense of devaluation that can result in shame, guilt, discrimination, and social unacceptability [6], along with adverse consequences such as psychosomatic issues [3]. Adverse outcomes include limited access to healthcare services and treatment, healthcare provider acceptance, resilience, and treatment adherence [7].

Most previous stigma studies have centered on patients with diverse conditions, including mental illness, HIV, AIDS, and, recently, cancer [4]. Studies about patients with cancer provide evidence of stigmatization within this population. In the report from the U.S. National Cancer Institute titled “Global Cancer Stigma Research Workshop,” it is highlighted that patients with cancer frequently experience fears of being blamed for their illness, held accountable for their cancer diagnosis, and facing potential abandonment by their social circles [8]. Many patients with lung cancer reported avoiding treatment to conceal their diagnosis [9,10]. Previous studies indicated that the public and healthcare providers held negative perceptions of patients diagnosed with lung cancer [11,12]. Furthermore, Else-Quest et al. [13] found that the specific type of cancer does not influence the experience of stigmatization.

Cultural norms and practices substantially influence a community’s lifestyle, beliefs, and values, affecting its overall well-being and ability to adapt and thrive [14]. The cultural belief system enables individuals to comprehend, interpret, explain, and label their experiences [15]. Furthermore, it shapes individuals’ responses to challenging and unforeseen circumstances [16]. People who share the same culture have certain values, beliefs, and practices that distinguish them from other cultures [17].

Oman is an Arab country with a predominantly Muslim population, situated in the Gulf region. In Oman, government healthcare facilities provide accessible medical services, either free of charge or at affordable rates, to both Omani citizens and expatriates working in the public sector. Due to the differences in social, economic, and religious beliefs between Arab and Western cultures, research findings from the West may not be directly applicable to individuals in Arab Muslim countries. For example, a study conducted in an Arabic Muslim country found that some cancer survivors preferred to isolate themselves to conceal their needs and the shame associated with being diagnosed with cancer [18]. Patients in Jordan associated cancer with death and hopelessness [19], and certain misconceptions about cancer were found to be associated with gender, education level, and income in this population [19]. Additionally, in Arabic communities, both healthcare providers and family members may view cancer as an evil or wicked disease [20]. Conversely, cancer survivors may experience self-stigmatization and emotional distress stemming from a perceived responsibility for their disease, with some blaming their lifestyle for their illness (5). Furthermore, it is common for healthcare providers and family members in Arab countries not to disclose a cancer diagnosis to patients [21]. The primary goal of this study was to explore the perceived stigmatization experienced by patients with cancer within the framework of Arabic and Islamic culture.

**Theoretical framework:** The Health Stigma and Discrimination Framework served as the foundational basis for this study, which elucidates how social judgment and attribution of blame can adversely affect health-related stigma experienced by Muslim patients diagnosed with cancer.

## Methods

### Design

A cross-sectional study was used to investigate the perceived stigmatization of Omani patients with cancer.

### Sample and settings

The study involved a sample of 190 participants, all recruited from two government-funded hospitals in Oman known for their expertise in cancer treatment. These hospitals serve as referral centers for patients with cancer referred from various regional hospitals across Oman. Recruitment took place from June 2021 to March 2023. The sample size for the study was determined using G Power V3.1 software for multiple regression analyses, with a significance level (α) of 0.05, a statistical power of 0.80, a medium effect size, and 10 predictors, resulting in a calculated sample size of 184. A medium effect size was assumed based on previous similar studies examining stigma levels among patients with chronic illnesses.

The study included participants aged 18 or older, diagnosed with cancer, aware of their diagnosis, and proficient in understanding Arabic. Participants were ineligible if they had been diagnosed with cancer in the three months preceding data collection, were hospitalized during the study, or had significant psychiatric issues that could impact questionnaire completion. The minimum age of 18 was chosen to focus on the experiences of adult patients with cancer. Newly diagnosed patients may face heightened challenges during the initial three months, which can significantly impact their experiences.

### Instruments

#### Social Impact Scale (SIS).

The 24-item self-report scale is multidimensional and comprises four domains: isolation (7 items), social rejection (9 items), internalized shame (5 items), and financial security (3 items) [22]. Participants respond to scale items using a four-point Likert-type scale, ranging from “strong disagreement” to “strong agreement.” The total score is obtained by summing all four subscale scores, with the highest possible score being 96. Higher scores indicate a greater level of stigmatization. This scale has demonstrated satisfactory to excellent psychometric properties among patients with cancer and AIDS. The internal consistency coefficients for the subscales ranged from.85 to.90, accompanied by low correlations between subscales [22]. The Brislin (1986) model of translation was employed in this study to convert the English version into Arabic through both forward and backward translations by bilingual team members and a doctoral-prepared translator, ensuring accuracy and cultural relevance. Two bilingual reviewers assessed the face validity of the Arabic version for clarity and consistency, while an Arabic monolingual layperson evaluated it for simplicity and broader audience understanding [23] .

**Demographic form:** This form was utilized to gather information about all participants, including their age, gender, marital status, educational background, employment status, and monthly income.

**Medical form:** This form was used to collect the following information: time since diagnosis, type of cancer, cancer stage, types of treatment, treatment completion status, family history of cancer, other existing health conditions (e.g., mental illness and HIV/AIDS), and smoking habits.

#### Ethical approval.

Ethical approval was obtained from the Institutional Review Board of the College of Nursing at Sultan Qaboos University. The Helsinki Declaration (1964) and its subsequent amendments guided all the procedures performed. Informed consent forms were obtained from all the subjects prior to their participation in the study.

### Study procedure

The study was granted ethical approval by the Research Ethics Committees of both the academic institution and the two research settings. The nursing staff informed the patients about the study, and the research team subsequently approached those who showed interest in participating and met the eligibility criteria. They were provided with a comprehensive explanation of the study, including its purpose, potential risks, benefits, and procedures, and were asked to consent to participate. Upon obtaining written consent, participants completed the study questionnaires at home or in a designated quiet area at the hospital. Furthermore, the research team retrieved medical information from the participants’ records.

### Data analysis

The data was analyzed using SPSS (version 24.0; SPSS Inc., Chicago, IL). A significance level (alpha) of p < .05 was set. Descriptive statistics, including means, standard deviations, and ranges, were calculated for the four subdomains of perceived stigma (social isolation, social rejection, financial insecurity, and internalized shame) and the total scores to characterize perceived stigmatization among patients with cancer.

## Results

### Sociodemographic and medical characteristics

Out of 194 patients invited to participate in the study, 190 consented to take part, resulting in a response rate of 97%. This high level of participation enhances the reliability of the study’s findings. The average age of the participants was 46.52 years (SD = 13.9). Approximately 72% of the participants were female, and 73.6% were married. Nearly 40% of the participants held college or university degrees. Around 54.4% of the participants had a low income. Most participants (59.5%) were diagnosed with breast and colon cancers, were still undergoing cancer treatment (82.4%), and did not have other chronic diseases (67.9%). The average duration of cancer was 24.2 months (SD = 30.5), and the average treatment duration was 21.0 months (SD = 28.9) (Table 1).

**Table 1:** Socio-demographic and medical characteristics (N = 190).

Variables	N (%)	
**Sex**		
Male	54 (28.4)	
Female	136 (71.6)	
**Marital Status**		
Married	140 (73.7)	
Single	28 (14.7)	
Divorced	14 (7.4)	
Widowed	8 (4.2)	
**Family members**		
Less than 5	77 (40.5)	
6–10	71 (37.4)	
11–16	24 (12.6)	
**Education**		
Less than high school	110 (57.9)	
Higher than high school	80 (42.1)	
**Income**		
Low Income	107 (56.3)	
Moderate Income	51 (26.8)	
High Income	32 (16.8)	
**Employment status**		
Employed	63 (33.2)	
Not employed	127 (66.8)	
**Type of cancer**		
Breast	57 (30.0)	
Hematological	38 (20.0)	
Colon	32 (16.8)	
Others	63 (31.2)	
**Cancer Stage (I-IV)**		
I and II	70 (36.8)	
III and IV	87 (45.8)	
Hematological	33 (17.4)	
**Treatment completion**		
Completed	30 (16.8)	
Not completed	160 (84.2)	
Variables	Mean (SD)	Range
Age (year)	46.5 (13.9)	(19–85)
Duration of cancer (months)	24.2 (30.5)	(1–156)
Duration of treatment (months)	21.0 (29.0)	(1–156)

### The level of stigma

Table 2 presents the findings of a study on stigma. The average total stigma level was 82.36, which is considered high. The dimensions, ranked from highest to lowest, are as follows: social rejection (M = 3.69), financial security (M = 3.35), isolation (M = 3.34), and internalized shame (M = 3.16). The means for each subscale are detailed in Table 2. Furthermore, Table 3 illustrates the number of participants who responded to each item on the stigma scale, categorized by dimension.

**Table 2:** Descriptive statistics of the level of stigma (N = 190).

Tool	Mean (SD)	Actual Range	Possible Range	Cronbach’s Alpha
**Perceived stigma**				
Social rejection	32.21 (3.4)	21–36	9–36	85.9
Financial security	10.00 (2.1)	3–12	3–12	70.7
Internalized shame	15.78 (3.5)	8–20	5–20	72.5
Social isolation	23.37 (4.2)	12–28	7–28	82.5
Total Stigma	82.36 (10.3)	57–96	24–96	

**Table 3:** Frequency of stigmatization items among muslim Arab patients with cancer in Oman (N = 190).

Social Rejection	Strongly Agree	Agree	Disagree	Strongly Disagree
1. My employer/co-workers have discriminated against me	6	8	44	131
2. Some people act as though I am less competent than usual	4	17	52	117
3. I feel I have been treated with less respect than usual by others	1	2	27	160
4. I feel others are concerned they could ‘catch’ my illness through contact, like a handshake or eating food I prepare	0	3	36	151
5. I feel others avoid me because of my illness	1	4	33	152
6. Some family members have rejected me because of my illness	0	1	23	166
7. I feel some friends have rejected me because of my illness	0	3	26	161
8. I encounter embarrassing situations as a result of my illness	3	26	41	120
9. Due to my illness, others seem to feel awkward and tense when they are around me	4	10	40	136
**Financial Security**				
10. I have experienced a financial hardship that has affected how I feel about myself	11	20	51	108
11. My job security has been affected by my illness	21	31	24	96
12. I have experienced financial hardship that has affected my relationship with others	5	12	41	131
**Internalized Shame**				
13. I feel others think I am to blame for my illness	5	12	37	136
14. I do not feel I can be open with others about my illness	22	40	46	82
15. I fear someone telling others about my illness without my permission	25	37	40	88
16. I feel I need to keep my illness a secret	33	35	36	86
17. I feel I am at least partially to blame for my illness	13	28	45	104
**Isolation**				
18. I feel set apart from others who are well	5	15	42	128
19. I have a greater need than usual for reassurance that others care about me	14	42	53	81
20. I feel lonely more often than usual	11	27	48	104
21. Due to my illness, I have a sense of being unequal in my relationship with others	6	18	45	121
22. I feel less competent than I did before my illness	11	46	42	91
23. Due to my illness, I sometimes feel useless	4	12	54	120
24. My illness has affected my social relationship	7	27	47	109

## Discussion

The study findings indicate that Muslim Arab patients with cancer experience significant stigmatization. Among the identified dimensions, the highest stigmatization was observed in social rejection, while the lowest was noted in internalized shame. However, all dimensions demonstrated notably high levels of stigmatization.

This study demonstrates that patients with cancer experience significant social rejection from their social circles and immediate environments. In contrast, a study conducted in Germany found that individuals with various types of cancer reported low to medium levels of social rejection [24]. Additionally, research in China indicated that patients with leukemia encounter a medium level of social rejection [25]. Meanwhile, a study in the United States by Wood et al. [4] found that patients with prostate cancer reported lower levels of social rejection compared to those observed in our findings. The high level of social rejection experienced by Arab Muslim patients with cancer may be attributed to prevailing misconceptions surrounding cancer. In this study, patients reported high levels of social discrimination from some family members, friends, and the broader community due to their cancer. They felt less competent and respected. In Arabic communities, there is a tendency among some healthcare providers and some family members to perceive cancer through a lens of negativity, often considering it as an evil or wicked disease [20]. In Arab communities, cancer is often viewed as a punishment or a test from God. Moreover, it is frequently seen as an incurable disease that is closely linked to death [26]. These community views can negatively influence the experience of Muslim Arab patients with cancer [27]. Healthcare providers should thoroughly assess patients’ perceptions of community attitudes towards cancer. Additionally, they should make concerted efforts to enhance public awareness of cancer and its treatment options within these communities.

The level of stigmatization associated with internalized shame was significant; however, it ranked as the lowest among the different dimensions of stigma. Interestingly, the higher level of stigmatization related to social rejection did not exhibit the same negative internal consequences. Our findings are consistent with previous studies, which indicated that the levels of internalized shame among patients in Germany and China were relatively comparable to those observed in the participants from this study [24,25]. However, contrary to our findings, Wood et al. indicated that patients with prostate cancer in the United States reported lower levels of internalized shame. In Muslim patients with cancer, the lower levels of internalized shame, as compared to other dimensions of stigma, may be influenced by several factors. These factors include their belief in and connection to God, the social support received from family members, and a tendency to conceal their cancer diagnosis [28,29]. A qualitative study conducted by Alaloul et al. [28] found that Arab Muslim patients with cancer often choose to hide their diagnosis from the public to avoid negative comments and reactions from specific community members. In the current study, only one-third of the patients reported feeling comfortable disclosing their diagnosis and being open with others. These findings highlight the social stigma surrounding cancer and its impact on individuals’ willingness to share their health status. This perception can significantly influence various aspects of cancer care, including awareness, prevention, and treatment-seeking behaviors [30].

This study indicates that Muslim Arab patients diagnosed with cancer frequently experience profound feelings of isolation and loneliness stemming from stigma. These individuals often perceive themselves as distinct from those who are healthy, leading to a sense of inequality in their social relationships. Consequently, their illness contributes to feelings of worthlessness and inadequacy. In contrast to our findings, previous studies have indicated that patients with cancer in Germany, China, and the United States reported low to moderate levels of isolation attributed to stigma [4,24,25]. A literature review study found that women diagnosed with breast cancer in Arab countries employed isolation and prayer as strategies to mitigate the impact of the cultural stigma associated with cancer [27]. Healthcare providers are responsible for actively addressing the stigma associated with social isolation and its adverse effects on patients. Researchers need to identify targeted, culturally sensitive interventions that can enhance the well-being and health outcomes of individuals diagnosed with cancer.

## Conclusion

This study underscores the substantial issue of stigma faced by Arab Muslim patients diagnosed with cancer. The emotional and psychological challenges stemming from this stigma highlight the necessity for targeted support and interventions that address the unique needs of this population. Stigma is frequently intertwined with cultural factors that shape how cancer is perceived and discussed within different communities. Thus, culture plays a vital role in influencing perceptions and experiences of cancer, leading to varying levels of stigma associated with the disease across diverse cultural backgrounds.

### Implications

Stigma and its consequences present significant challenges for patients with cancer in Arab Muslim countries. To effectively tackle these issues, it is essential to implement comprehensive and collaborative actions. Evaluating stigma should be a core component of our ongoing assessment processes. Healthcare providers must enhance public education regarding cancer and its treatments while also raising awareness of the unique needs of patients with cancer. Researchers need to identify and develop culturally sensitive stigma-reduction interventions that aim to improve the well-being and health outcomes of individuals diagnosed with cancer. Furthermore, it is critical to conduct future studies that examine the longitudinal changes in stigma associated with cancer throughout the treatment process. Fostering an environment where patients feel comfortable expressing their emotions is vital. Additionally, policymakers should enact regulations to safeguard patients’ rights in the workplace. Developing culturally tailored stigma-reduction interventions.

### Study limitations

One notable limitation of this study is the absence of a scale to measure participants’ level of religiosity. Religiosity can influence perceptions of illness, coping mechanisms, and attitudes toward health-related stigma. Future research should incorporate a validated religiosity scale to enhance understanding of these dynamics and offer a more comprehensive perspective on the factors that shape these perceptions. The absence of multivariate analysis in our research limits our ability to identify independent predictors of stigma and hinders our capacity to adjust for potential confounding variables. Therefore, it is essential for future studies to implement multivariate models to effectively identify the predictors of stigma while adequately accounting for confounders.
